# Effects of weight-bearing exercise on a mini-trampoline on foot mobility, plantar pressure and sensation of diabetic neuropathic feet; a preliminary study

**DOI:** 10.1080/2000625X.2017.1287239

**Published:** 2017-02-20

**Authors:** Wararom Kanchanasamut, Praneet Pensri

**Affiliations:** ^a^ Interdisciplinary Program of Biomedical Science, Faculty of Graduate School, Chulalongkorn University, Bangkok, Thailand; ^b^ Department of Physical Therapy, Faculty of Allied Health Sciences, Chulalongkorn University, Bangkok, Thailand

**Keywords:** Diabetes, neuropathy, foot mobility, plantar pressure, sensation, foot ulcer

## Abstract

**Objective**: Foot and ankle exercise has been advocated as a preventative approach in reducing the risk of foot ulceration. However, knowledge about the appropriate types and intensity of exercise program for diabetic foot ulcer prevention is still limited. The current study aimed to examine the effects of an eight-week mini-trampoline exercise on improving foot mobility, plantar pressure and sensation of diabetic neuropathic feet.

**Methods**: Twenty-one people with diabetic peripheral neuropathy who had impaired sensation perception were divided into two groups. The exercise group received a foot-care education program plus an eight-week home exercise program using the mini-trampoline (*n* = 11); whereas a control group received a foot-care education only (*n* = 10). Measurements were undertaken at the beginning, at the completion of the eight-week program and at a 20-week follow-up.

**Results**: Both groups were similar prior to the study. Subjects in the exercise group significantly increased the range of the first metatarsophalangeal joint in flexion (left: *p *= 0.040, right: *p *= 0.012) and extension (left: *p *= 0.013) of both feet more than controlled subjects. There was a trend for peak plantar pressure at the medial forefoot to decrease in the exercise group (*p *= 0.016), but not in the control group. At week 20, the number of subjects in the exercise group who improved their vibration perception in their feet notably increased when compared to the control group (left: *p *= 0.043; right: *p *= 0.004).

**Conclusions**: This is a preliminary study to document the improvements in foot mobility, plantar pressure and sensation following weight-bearing exercise on a flexible surface in people with diabetic neuropathic feet. Mini-trampoline exercise may be used as an adjunct to other interventions to reduce risk of foot ulceration. A larger sample size is needed to verify these findings. This trial is registered with COA No. 097.2/55.

## Introduction

Diabetes mellitus is a non-communicable disease which is a national and global public health problem. It is estimated that over 360 million people worldwide will have the disease by year 2030.[1] In developing countries, the prevalence of diabetes has been increasing steadily. A survey of the number of people with diabetes in Thailand in 2003 found that approximately 2.4 million people were living with diabetes and about half of them did not know that they had the disease.[2] Diabetes mellitus leads to many complications. A common complication of diabetes is diabetic peripheral neuropathy (DPN). The symptoms of DPN vary; however, loss of sensation in one or both feet is often an initial sign. Therefore, people with diabetic peripheral neuropathy are inclined to have foot ulcers and are at high risk of foot or leg amputation.[3] Additionally, other causes may lead to foot ulceration such as limited joint mobility and high plantar pressure.[4] Foot ulcer due to diabetes mellitus is a major health problem since it is related to quality of life of the patient and the source of enormous costs to healthcare services.[5,6] The most costly and feared consequence of a foot ulcer is limb amputation,[7,8] which occurs 10 to 30 times more often in people with diabetes than in the general population.[9–11] The estimated cost of treating a diabetic amputee is $34,000 per year.[12] The American Diabetes Association (ADA) has suggested that the best practices in lower limb amputee prevention are reducing risk of foot ulceration and providing appropriate foot care education to patients.[10,13] Moreover, proper foot and ankle exercise has been advocated as another imperative preventative step to reduce the risk of foot ulceration and amputation.[14] Since an increase in weight-bearing activity can lead to higher plantar pressure, resulting in the foot ulceration, the ADA recommends people with DPN to suitably perform the exercise with limited weight-bearing activities.[15–17]

There is limited evidence about the use of exercise programs to decrease the risk of foot ulceration among people with DPN. Previous studies have shown conflicting findings. For example, Flahr [13] applied a non-weight-bearing ankle exercise regimen to people with DPN and foot ulcers and showed that the exercise did not provide better wound reduction than usual care, but could increase joint mobility and improve blood circulation. Goldsmith et al. [18] studied the effect of a home-based active and passive range-of-motion exercise program on joint mobility and plantar pressure for people with diabetes. They found that a range-of-motion exercise program could reduce peak plantar pressure and thus might reduce the risk of foot ulceration.[18] LeMaster et al. [19] studied the effects of an individually adapted weight-bearing exercise program on physical activity and incidence rate of foot lesions among people with DPN, and found that the total incidence of foot lesions and ulcers did not significantly differ between groups. However, Balducci et al. [15] studied long-term brisk walking on a treadmill and reported that such an exercise intervention could improve the hallux vibration perception threshold of people with diabetes.

There was no conclusive evidence regarding the types of exercise that were useful for diabetic foot ulcer prevention. Further research in this area is therefore needed. A previous study has shown that weight-bearing exercise on an elastic surface can help to reduce contact pressure between the foot and ground.[20] Recently, the benefits of trampoline exercise were presented in some specific subject groups including improved balance control among older adults,[17] decreased cervical muscle spasm in fighter pilots,[16] and improved foot function in athletes with ankle instability.[21] To our knowledge, research studies examining the effects of trampoline exercise on foot care are unavailable. According to the evidence, exercise on a mini-trampoline promotes muscle strength, balance, and joint mobility. Therefore, we anticipated that exercise on a mini-trampoline might help patients with DPN to prevent foot ulceration by reducing neuropathic symptoms and high plantar pressure, as well as increasing foot and ankle strength. This study aimed to investigate the effects of a mini-trampoline exercise program on increasing foot mobility, decreasing peak plantar pressure, and enhancing sensation perception of people with DPN.

## Methods

**Figure 1.**
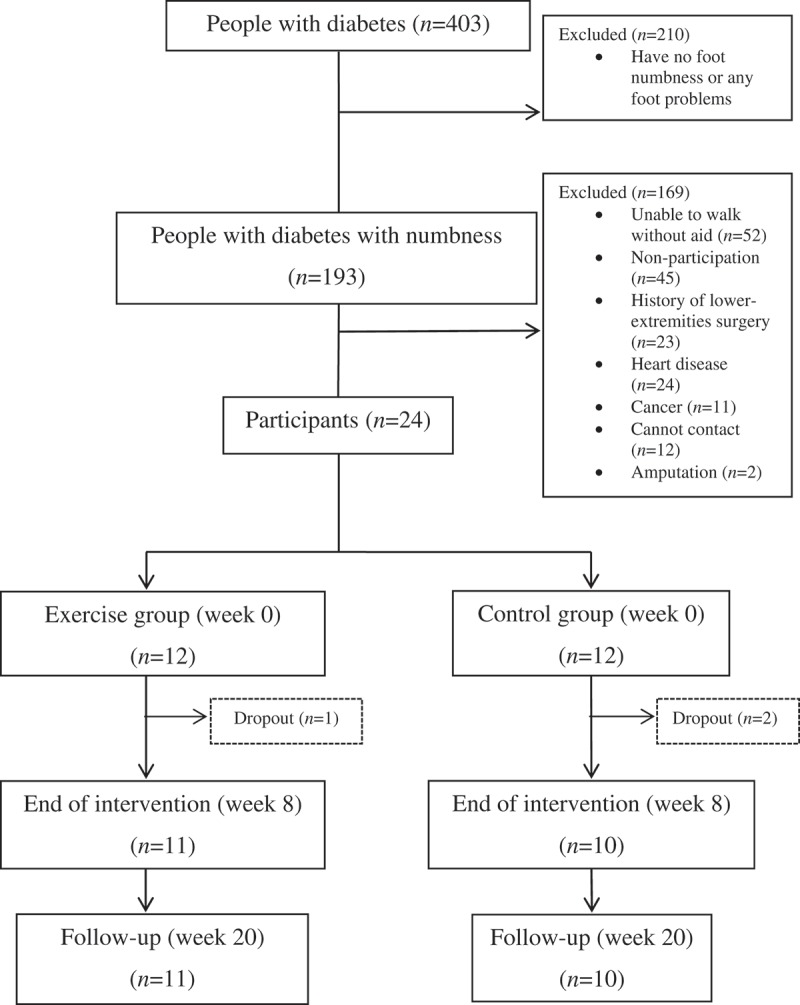
Flowchart of participants.

The present study was a controlled clinical trial that aimed to compare changes in DPN symptoms among diabetic patients with and without using weight-bearing exercise on a mini-trampoline (Figure 1). The exercise intervention continued for eight weeks. Prior to the intervention, we collected each subject’s demographic data, including age, weight, body mass index (BMI), duration of diabetes mellitus, duration of foot numbness, and underlying symptoms. To investigate treatment outcomes we held three measurement sessions: the measurement of flexion and extension angles of the first (1st) metatarsophalangeal joint (MTPJ); the evaluation of plantar pressure distribution; and sensory perception testing. We recorded treatment outcomes at the beginning of the program (week 0), at the end of the exercise program (week 8), and at the end of the follow-up period (week 20) using a single blinded outcome assessor for the groups of subjects. Subjects in the exercise group were given a supervised exercise program on a mini-trampoline by a senior physical therapist.

Initially, a total of 403 Thai patients expressed interest in participating in our study. They were diabetic patients from the respective Diabetes Clubs of four healthcare facilities: Chulalongkorn Memorial Hospital, Bangkok; Phramongkutkloa Hospital, Bangkok; and Bangjak and Bangkru Sub-District Primary Care Unit, Ladluang District, Samutprakarn Province, Thailand. After primary screening, 193 patients with diabetes mellitus-related foot problems met the inclusion criteria. The inclusion criteria consisted of a medical diagnosis of DPN with loss of feeling in the foot and BMI equal to 18–30 kg m^–^^2^. Subjects were excluded if they had at least one of the following conditions: malignancy; myocardial infarction; stroke; hepatic failure; renal failure/dialysis; angina; embolism; cardiac arrhythmia; previous bypass surgery/angioplasty; foot/leg amputation; current or previous foot ulceration; reduced palpability of dorsalis pedis and tibialis posterior arteries; and participation in regular weight-bearing exercise, such as walking, running or foot exercise. Finally, 24 patients met the criteria and were eligible for entry into the study, as shown in Figure 1. They were allocated using convenience sampling method into two groups: 12 subjects in an exercise group and the other 12 in a control group. However, one subject dropped out from the exercise group prior to the end of week 8 due to flu; and two subjects dropped out of the control group for unknown reasons; they could not be contacted.

This study was undertaken between April 2013 and January 2015. Approval to undertake the study was obtained from the Ethics Committee for Research Involving Human Research Subjects, Health Science Group, Chulalongkorn University, Thailand (COA No. 097.2/55).

### Outcome measurements

The flexion and extension angles of the 1st MTPJ were measured using a passive range of motion (ROM) measurement technique. A stainless steel goniometer was used to measure the joint motion. Stationary and movable arms of a goniometer were placed on the first metatarsal and the proximal phalanx of the hallux, respectively. Each subject was asked to sit with straight legs. The assessor held the stainless steel goniometer and first metatarsal with one hand and moved the proximal phalanx of the hallux with movable arm with the other hand. To measure the 1st MTPJ’s passive maximum flexion angle, the assessor moved the subject’s big toe caudally. To measure the 1st MTPJ’s passive maximum extension angle, the assessor moved the subject’s big toe towards the subject’s head. Data were recorded as left 1st MTPJ flexion, right 1st MTPJ flexion, left 1st MTPJ extension, and right 1st MTPJ extension. The mean of three maximum flexion and maximum extension recordings was calculated for analysis.

Footscan® 7 hardware (RSscan International NV, Paal, Belgium) was used to measure and the software was used to record and analyze the plantar pressures. Initially, each subject was asked to walk barefoot on the platform for familiarity. The platform, of which dimensions were 2096 mm × 472 mm × 18 mm, was calibrated using the subject’s body weight before each testing session. Then the subject was asked to walk naturally, and barefoot, on the platform three times. The average value of the three peak plantar pressure measurements was used for data analysis. Plantar areas were divided into five areas for analysis, including areas under hallux, medial forefoot, lateral forefoot, midfoot and heel.[22]

Sensory deficit was assessed to determine the impairment of pressure and vibration perception. Pressure perception was tested using 10 g Semmes-Weinstein monofilament (Amaryl®, Sanofi Aventis U.S. LLC, NJ, USA) at the plantar aspect of the 1st, 3rd and 5th metatarsal heads and plantar surface of the distal hallux. Subjects with the pressure perception deficit were those who lost sensation in at least one site.[23] First, the assessor showed the monofilament to the subject and applied it to the upper arm to demonstrate the sensation. Then, the assessor instructed subjects to say ‘yes’ if they perceived the monofilament sensation. Subjects were asked to close their eyes during the test. Furthermore, the assessor used false tests that asked subjects if they could feel the monofilament when it was not being applied. Vibration perception was tested using a 128 Hz tuning fork (Spirit®, Spirit Medical Co., Ltd., New Taipei City, Taiwan) that was placed over the pulp of the hallux. Subjects with a vibration perception deficit were those who abnormally detect the cessation of the tuning fork when the assessor still perceived it. First, the assessor knocked the tuning fork against her palm and placed it over the subject’s sternum to demonstrate the vibration. Further, subjects were instructed to tell the assessor when they felt the cessation of vibration.

In addition, symptoms of reduced/lost feeling in the feet were assessed using the neuropathy and foot ulcer-specific quality of life instrument (NeuroQoL, Manchester Royal Infirmaty, Manchester, UK) as a subjective evaluation. The NeuroQoL instrument was used to ask patients with DPN about their diabetic peripheral neuropathy-related symptoms and psychosocial functioning in seven domains.[24] The current study used the ‘symptom of reduced/lost feeling in the feet’ domain, which contained three items, to record the impairment of patients’ sensation. An individual patient rated their symptoms of reduced/lost feeling in the feet on a five-point Likert scale (never, occasionally, some of the time, most of the time and all the time). An average score obtained from the scores of three items represented this specific domain of the NeuroQoL.[24]

### Intervention

The current home-based mini-trampoline exercise program was adapted from two sources: a previous study by Aragão et al. [17] and a foot exercise program presented in a standard care booklet for people with diabetes. Aragão et al. [17] applied a mini-trampoline exercise program to improve balance among elderly people without underlying disease. The program contained various vigorous weight-bearing activities such as jumping and hopping. The mini-trampoline exercise program used in the current study was easier to perform than that used in Aragão’s study, because diabetics with nerve dysfunction normally had poor balance compared to healthy elderly people.[5] It was thus necessary to avoid risk of injury and fall by deleting some difficult performances such as jumping and hoping. Modification of the remaining actions was carried out, including changing from running in place to walking in place on the mini-trampoline, changing from a one-foot jump with front and back displacement to a two-feet alternae jump with front and back displacement. The exercise intensity was gradually increased by omitting simple activities and adding more complex activities into the program. It was expected that various exercise activities used in the designed program would increase leg and foot muscle contractions without aggravating foot complications. Moreover, in order to promote flexibility of joints in the ankle and foot, some motions such as ankle inversion/eversion of both feet, and toe raised with both legs standing were added as seen in Table 1. A pilot study was carried out to examine the practicality and ease of use, as well as the safety of a mini-trampoline for use at home.

**Table 1.** Exercise program on mini-trampoline.

Level 1	Level 2	Level 3	Level 4
- Both legs standing - March walking - One leg standing - Toe raises with both legs standing - Step forward and backward slowly	- One leg standing - March walking - Each ankle inversion and eversion - Step forward and backward slowly - Step sideways - Lightly jump with both legs	- One leg standing - Standing, cross legs at ankles - Toe raises, on one foot - Step sideways - Move opening and closing legs - Lightly jump sideways with both legs - Both ankle inversion and eversion	- Standing, cross legs at ankles - Heel stand, on one foot - Canter - Step sideways - Alternate lightly jumping forward and backward - Both legs standing with closed eyes - Ankle inversion and eversion, on one foot - Heel stand, on one foot

The present controlled clinical trial applied a single blind measurement to the groups of subjects when collecting data. Subjects in the exercise group participated in the exercise program on a mini-trampoline and received foot-care knowledge, whereas those in the control group only joined the usual foot-care education for people with diabetes. Each subject in the exercise group was given a supervised exercise program by a physical therapist. The training materials included a demonstration DVD, a mini-trampoline exercise booklet diary and a mini-trampoline, 100-cm spring steel trampoline model Trimilin® Med (100 kg load capacity; TÜV product service, Suzhou High-Ten Sports Equipment Co., Ltd, Jaingsu, China).

For each level of the exercise program, the subject was asked to exercise on a mini-trampoline at least five times per week for two consecutive weeks at home. The exercise program consisted of four levels of progression for a total period of eight weeks. To warm up prior to exercise and cool down afterwards, subjects were instructed to stretch their quadriceps femoris, hamstring and gastrocnemius, and soleus muscles. A stretch of each muscle was held for 20 s and repeated three times. Then, the subjects were instructed to carry out different exercise positions with 10 repetitions each and were asked to hold static exercise positions for 10 s. The prescribed exercise program each day was three sets with five-minute rest intervals between sets. They were asked to wear proper footwear which they received from the researcher. Additionally, foot checking was required to be carried out after exercise. Finally, subjects were given a mini-trampoline exercise booklet diary to record their exercise frequency. To maintain patient compliance with the program, subjects were regularly contacted and encouraged by the researcher for their exercise involvement by telephone.

All subjects from both groups received a foot care booklet for people with diabetes. The booklet contained basic knowledge about foot-care, including prevalence and incidence of foot ulcers and amputation, risk factors, and instructions for foot self-care. Apart from providing foot care education, the booklet also served as a personal diary in which the subjects could record their self-foot care activities, as required in the study.

### Data analysis

#### Sample size calculation

The sample size was calculated from the data of the 1st MTPJ ROM obtained from our pilot study about the effect of mini-trampoline exercise among eight patients with DM. A prior power calculation was based on an effect size of 0.6 (mean of pre: 26.26, mean of post: 31.98 and SD: 9.11), a significant level of alpha at 0.05 and a power value of 95% was obtained via GPower (version 3.0.10, www.gpower.hhu.de). The sample size of 10 participants per group was estimated. Allowing for a dropout rate that did not exceed 20% of sample size, the minimum sample size required was 12 participants per group.

### Statistical analysis

Statistical analysis was conducted using the Statistical Package for the Social Sciences (SPSS) software (version 17.0, SPSS Inc., Chicago, IL, USA). Frequency of gender and the number of subjects who had lost or reduced pressure and vibration perception, were calculated. Mean and standard deviation of continuous data were used; these included age, BMI, weight, height, duration of diabetes, duration of DPN, 1st MTPJ ROM, peak plantar pressure, and score for the ‘symptom of reduced/lost feeling in the feet’ domain of NeuroQoL. Shapiro–Wilk test was used to determine normal data distribution. The current results showed normal distribution of all data except age and duration of peripheral neuropathy.

In order to compare the significant difference of outcome measures collected at baseline, week 8, and week 20, the following analyses were computed. With regards to the change of pressure perception and vibration perception within each group, McNemar’s test was used, while Mann–Whitney U test was used for the comparison between groups. For continuous data, a mixed-design repeated measures analysis of variation (mixed-ANOVA) was performed to determine main effects and interaction effects of independent factors on dependent variables. Intervention (i.e. exercise group and control group) was a between-subject factor. Time of data collection (i.e. baseline, week 8, week 20) was a within-subject factor. The 1st MTPJ ROM, peak plantar pressure and symptoms of reduced/lost feeling in the feet domain of NeuroQoL score were dependent variables. Pairwise comparisons were implemented using Bonferroni test to identify direction of dependent variables. The significant level was set at 0.05.

## Results

### Baseline characteristics

Demographic data of 11 subjects in the mini-trampoline exercise group and 10 subjects in the control group are shown in Table 2.

**Table 2.** Demographic data of exercise group and control group.

	Exercise group (*n* = 11)		Control group (*n* = 10)		
Variables	Mean ± SD	Min. – Max.	Mean ± SD	Min. – Max.	*P*-value
Age (years) *	64.8 ± 7.4	53 – 72	65.1 ± 7.7	51 – 75	0.933
BMI (kg m^–^^2^) ^†^	24.2 ± 2.3	21.2 – 29.8	25.3 ± 2.3	22.0 – 28.4	0.279
Weight (kg) ^†^	58.5 ± 8.5	45.5 – 79.1	61.1 ± 9.9	48 – 75	0.530
Duration of diabetes (years) ^†^	14.6 ± 7.6	5 – 26	14.3 ± 8.0	6 – 32	0.896
Duration of peripheral neuropathy (years) *	4.0 ± 4.5	1 – 15	4.2 ± 4.9	1 – 15	0.923

*Mann–Whitney U test (non-normal distribution).

^†^Independent sample *t*-test (normal distribution).

Values are means ± standard deviation (SD).

Min., minimum; Max., maximum.

### First metatarsophalangeal joint movement

A mixed-ANOVA test revealed the difference in the 1st MTPJ ROM between the exercise and control groups. The test result showed that there was a statistically significant interaction between the intervention and time on the left 1st MTPJ flexion: F(2,18) = 5.427, *p *= 0.008, right 1st MTPJ flexion; F(2,18) = 7.175, *p *= 0.002. At week 20, 1st MTPJ flexion showed a significant increase that was greater in the exercise group compared to the control group (left: 46.69 ± 14.48 vs. 34.23 ± 11.01; *p *= 0.040, right: 41.88 ± 13.69 vs. 27.47 ± 9.47; *p *= 0.012). Similarly, there was a statistically significant interaction between intervention and time on the left 1st MTPJ extension, F(2,18) = 4.490, *p *= 0.018, and right 1st MTPJ extension, F(2,18) = 8.475, *p *= 0.001 and time main effect, F(2,18) = 6.773, *p *= 0.003. The 1st MTPJ extension showed a significant increase that was greater in the exercise group compared to the control group, as found in the left foot (57.64 ± 7.32 vs. 46.17 ± 11.66; *p *= 0.013), as seen in Table 3. Within-group comparisons of the exercise group, on three occasions, showed that there was a significant difference in left and right 1st MTPJ for both flexion and extension when data were compared between week 0 and week 20 (left flexion: 26.67 ± 11.18 vs. 46.69 ± 14.48; *p *= 0.00, right flexion: 26.27 ± 8.55 vs. 41.88 ± 13.69; *p *= 0.00, left extension: 42.73 ± 15.92 vs. 57.64 ± 7.32; *p *= 0.005, right extension: 38.06 ± 10.34 vs. 51.48 ± 9.16; *p *= 0.004), week 0 and week 8 (right flexion: 26.27 ± 8.55 vs. 39.27 ± 18.08; *p *= 0.032, right extension: 38.06 ± 10.34 vs. 47.45 ± 11.93; *p *= 0.09), as well as week 8 and week 20 (left flexion: 26.67 ± 11.18 vs. 39.33 ± 17.26; *p *= 0.038) as seen in Table 3.

**Table 3.** Comparison of mean and standard deviation of 1^st^ metatarsophalangeal joint range of motion and peak plantar pressure.

	Exercise group (*n* = 11)			Control group (*n* = 10)		
Variables	Week 0	Week 8	Week 20	Week 0	Week 8	Week 20
Flexion of 1^st^ metatarsophalangeal joint (degrees)						
Left foot	26.67 ± 11.18^‡^	39.33 ± 17.26^§^	46.69 ± 14.48^‡§^*	32.67 ± 9.42	31.13 ± 12.65	34.23 ± 11.01*
Right foot	26.27 ± 8.55^†‡^	39.27 ± 18.08^†^	41.88 ± 13.69^‡^*	31.23 ± 5.54	28.53 ± 9.88	27.47 ± 9.47*
Extension of 1^st^ metatarsophalangeal joint (degrees)						
Left foot	42.73 ± 15.92^‡^	52.48 ± 14.44	57.64 ± 7.32^‡^*	48.97 ± 11.53	48.03 ± 15.78	46.17 ± 11.66*
Right foot	38.06 ± 10.34^†‡^	47.45 ± 11.93^†^	51.48 ± 9.16^‡^	47.27 ± 13.71	42.87 ± 15.33	43.93 ± 13.96
Peak plantar pressure of left foot (N cm^–^^2^)						
Hallux	45.72 ± 19.40	48.36 ± 26.23	46.51 ± 14.83	35.28 ± 14.18	36.09 ± 17.92	38.39 ± 24.22
Medial forefoot	24.98 ± 14.42	28.87 ± 17.37	27.37 ± 17.87	23.73 ± 15.98	22.05 ± 11.16	21.68 ± 15.68
Lateral forefoot	49.03 ± 8.36^‡^	49.76 ± 10.44^§^	58.21 ± 11.18^‡§^	33.81 ± 10.31	37.42 ± 22.11	40.62 ± 22.72
Midfoot	20.42 ± 3.59	23.38 ± 7.53	25.33 ± 7.95	19.11 ± 7.23	19.22 ± 5.95	23.69 ± 11.76
Heel	35.42 ± 7.14	42.00 ± 9.51	40.74 ± 7.80	40.23 ± 12.85	42.37 ± 12.01	42.31 ± 12.32
Peak plantar pressure of right foot (N cm^–^^2^)						
Hallux	35.59 ± 13.23	35.16 ± 17.65	38.10 ± 27.91	29.22 ± 14.20	36.71 ± 15.35	34.12 ± 15.08
Medial forefoot	39.56 ± 15.25^‡^	42.18 ± 16.14^§^	31.46 ± 12.94^‡§^	36.17 ± 17.37	40.39 ± 18.26	34.03 ± 12.84
Lateral forefoot	49.47 ± 8.98	51.29 ± 11.68	48.43 ± 15.35	40.17 ± 12.97	45.17 ± 23.09	43.82 ± 23.98
Midfoot	19.31 ± 2.14	21.15 ± 11.05	20.91 ± 9.27	17.89 ± 5.59	18.57 ± 7.89	17.07 ± 5.49
Heel	33.49 ± 7.73	37.06 ± 8.43	38.17 ± 8.57	33.14 ± 15.99	38.56 ± 18.85	40.08 ± 24.33

*Significant difference between groups.

^†^Significant difference between week 0 and 8 in exercise group.

^‡^Significant difference between week 0 and 20 in exercise group.

^§^Significant difference between week 8 and 20 in exercise group.

### Peak plantar pressure

Means and standard deviations of peak plantar pressure for five areas in the plantar aspect of the foot, including hallux, medial forefoot, lateral forefoot, midfoot and heel, are shown in Table 3. The results showed that there was no statistically significant interaction between the intervention and time on peak plantar pressure. However, within-group comparisons found that there was a significant decrease in peak plantar pressure of the right exercising foot at the medial forefoot at week 20 (31.46 ± 12.94) compared to week 0 (39.56 ± 15.25) (*p *= 0.016). With respect to the left exercising foot there was a significant increase in peak plantar pressure in lateral forefoot at week 20 (58.21 ± 11.18) compared to week 0 (49.03 ± 8.36) (*p *= 0.034). Conversely, there was no significant change of peak plantar pressure in both feet of subjects in the control group.

### Sensation perception

According to the monofilament testing, all subjects had lost or reduced pressure and vibration perception at the beginning. After finishing the exercise program, subjects in the exercise group could improve their somatosensory perception. There were 36.4% of subjects in the exercise group (*n* = 4) who still had pressure perception deficits at week 8 and 45.5% (*n* = 5) at week 20. Likewise, there were 36.4% of subjects in the exercise group (*n* = 4) who still had deficits in vibration perception at week 8, and 18.2% (*n* = 2) at week 20.

Using McNemar’s test for comparing within-group data at baseline, week 8 and week 20, the number of subjects who had deficits in pressure perception in the exercise group significantly reduced for both feet (*p *= 0.008–0.031), whereas the number of subjects in the control group did not significantly change. Similarly, the number of subjects who had deficits in vibration perception in the exercise group significantly decreased for both feet (*p *= 0.004–0.016), whereas the number of subjects in the control group did not significantly decrease.

There was a statistically significant difference in the number of subjects who had lost or reduced pressure perception of the left foot at week 8 between the exercise and control groups (*p *= 0.013), as shown in Table 4. There was a statistically significant difference in the number of participants who had lost or reduced vibration perception of the right foot at week 8 (*p *= 0.013), and both feet at week 20 (left: *p *= 0.043; right: *p *= 0.004), as shown in Table 4.

**Table 4.** Comparison of lost or reduced pressure and vibration perception between groups.

	Exercise group (*n* = 11)			Control group (*n* = 10)		
	Week 0	Week 8	Week 20	Week 0	Week 8	Week 20
Pressure perception						
Left foot	11	3*	5	10	9*	8
Right foot	11	4	5	10	8	9
Vibration perception						
Left foot	11	4	2*	10	8	7*
Right foot	11	3*	2*	10	9*	9*

*Significant difference between groups.

Using repeated measure analysis of variance, a statistically significant interaction between the intervention and time effect on ‘symptoms of reduced/lost feeling in the feet’ domain of the NeuroQoL was found; F(2,18) = 6.239, *p *= 0.004. This result was in line with the findings of the monofilament testing. There was a significant decrease in the NeuroQoL score regarding symptoms of reduced/lost feeling in the feet in the exercise group when comparing week 0 (2.58 ± 0.78) with week 8 (1.85 ± 0.82) (*p *= 0.004), and week 0 (2.58 ± 0.78) with week 20 (1.91 ± 0.68) (*p *= 0.003), while there was no statistically significant decrease in the NeuroQoL score in the control group.

## Discussion

The aim of this study was to compare the change in range of motion of the 1st MTPJ, peak plantar pressure, and sensation perception between the mini-trampoline exercise group and control group in people with DPN.

From our best knowledge, this is the first study to investigate the effect of a foot exercise program in people with DPN by standing and dragging the foot on an elastic surface. ADA suggests that people with DPN should limit weight bearing exercise to decrease the risk of foot ulceration. The current study demonstrated that weight-bearing exercise on a mini-trampoline could enhance sensation perception and increase foot mobility in people with DPN.

Previous studies have demonstrated that exercise programs using a mini-trampoline improve balance ability in various population groups, including the elderly,[17] stroke patients,[25] and children with intellectual disabilities.[26] It has been documented that exercises on a mini-trampoline involve a multi-component approach, including muscle coordination, strength and balance training, body stability, and joint flexibility training.[25] The benefits of exercises on a mini-trampoline could be obtained within three weeks of training for stroke patients to improve postural control and activities of daily living,[25] and within 14 weeks for the elderly to regain balance.[17] The authors suggested that the improvement in mobility and balance might be related to improved plantar flexor muscle strength and hip moment generation.[17] Muscle action and coordination in the lower extremities were continuously facilitated by maintaining body balance on an elastic surface. In general, the ability to maintain balance is based on three mechanisms, including increasing the base of support, counter-rotating segments around the center of mass, and applying an external force other than the ground reaction force.[27] In performing the mini-trampoline exercise, the participants were challenged to stabilize their body while keeping the center of mass over the base of the support.[25] They needed to exert muscle force and neuromuscular responses to stiffen their legs in order to overcome the unstable conditions.[28]

Different surface hardness can have an impact on motor and perceptual change.[28] When walking or jumping, the human musculoskeletal system can modify its stiffness in response to the physical characteristics of the surface.[29] Improved leg stiffness due to exercise on an elastic surface could decrease the average muscle force required for exercise activity by increasing the external force produced by the elastic surface.[28] The elastic surface of the mini-trampoline in the current study was made from polypropylene; such material could support and distribute plantar pressure while subjects were standing and walking on its surface. As a result, subjects could improve their dynamic stability after the exercise training program. In addition, elasticity of the surface could increase the flexibility of foot and ankle joints during movement on elastic and unstable surfaces.[30]

This clinical trial was the first study to investigate mini-trampoline equipment as an option for reducing the risk of foot ulceration, consistent with the ADA recommendation. The specific foot exercise program for people with DPN on a mini-trampoline in this study was adapted from the study of Aragão et al. [17]. The previous study investigated the ability to regain balance in the healthy elderly, whereas this study focused on elderly people with DPN. Normally, elderly people with DPN had nerve dysfunction leading to a decrease in ability to control balance.[5] For safety, the exercise activities designed for this study were less difficult than those of the previous study.

Motor neuropathy in diabetes can affect intrinsic muscle atrophy that subsequently causes foot deformity and limited joint range of motion. A decrease in ankle and 1st MTPJ mobility can cause high plantar pressures under the metatarsal heads and loss of toe function, especially of the big toe.[4,31] The current study illustrated that exercise on an elastic surface could enhance the flexibility of the 1st MTPJ ROM and change plantar pressure distribution.

There was no previous study concerning the change in the 1st MTPJ ROM among people with DPN after receiving an exercise program. The improvement of the 1st MTPJ ROM in the exercise group might be explained by the fact that there was active stretching of joint ligaments and muscular tendons in ankles and toes, in combination with stretching of the plantar fascia during standing and walking on the elastic surface, resulting in the increased degrees of 1st MTPJ flexion and extension. Since the limitation of 1st MTPJ ROM can result in an abnormal gait pattern, especially in the propulsive phase, the improvement of 1st MTPJ ROM might promote better gait patterns among people with DPN in current study. Post-hoc analysis was undertaken to compare changing direction of the 1st MTPJ ROM. The analysis indicated that weight-bearing exercise on a mini-trampoline could improve mobility of the 1st MTPJ, which was the main joint when humans walk in the toe-off phase of a normal gait cycle.

The within-group analysis of peak plantar pressure distribution in the exercise group showed that patients with DPN had lower peak plantar pressure at the medial forefoot and higher peak plantar pressure at the lateral forefoot after completing the eight-week mini-trampoline exercise. Decreased medial forefoot peak plantar pressure might be related to an increase in the 1st MTPJ ROM. This notion was supported by the results of the study of Fernando et al. [32]. The authors revealed that different degrees of joint ROM had an effect on the changes in peak plantar pressure.[32] Similarly, Goldsmith et al. [18] reported a high positive correlation between an increase in the 1st MTPJ ROM and peak plantar pressure change among patients with diabetes. Obviously, the flexibility of the 1st MTPJ could promote the adjustment of peak plantar pressure.[18]

According to the literature, DPN patients commonly had high peak plantar pressure at their forefoot area, especially at the first metatarsal head or medial forefoot.[33] Primarily, it was expected that the mini-trampoline exercise program would reduce peak plantar pressure at medial forefoot. A decrease in medial forefoot plantar pressure, in association with an increase in lateral forefoot plantar pressure during walking, might indicate that subjects could shift their body weight to the lateral forefoot aspect more easily post-exercise than they could before performing the exercise. Since peak plantar pressure of the forefoot was directly correlated with diabetic neuropathy symptoms and could predict foot ulceration,[4] the change of peak plantar pressure distribution might reduce the risk of foot ulceration at medial forefoot of the exercise group. In contrast to the decreased plantar pressure at the medial forefoot, increased plantar pressure at the lateral forefoot did not increase the risk of ulceration. Normally, weight bearing during stance phase is transferred from heel, midfoot, lateral forefoot, medial forefoot and hallux, respectively,[34] of which the heel and big toe were the common plantar high load areas during heel strike and toe-off phase. Accordingly, an increase in plantar pressure at the lateral forefoot might suggest the adjustment of a subject’s gait pattern toward the normal gait cycle, especially in the stance phase. Furthermore, peak plantar pressure in this study did not exceed 65 N cm^–^^2^; such a level was considered as abnormal peak pressure and was indicative of diabetic foot risk factor.[35]

An increase in pressure and vibration perception of people with DPN after the completion of the mini-trampoline exercise program together with an improvement of reduced/lost feeling in the feet might result from peripheral microvascular dilation.[36] Weight-bearing and dynamic body movement on the mini-trampoline required the subjects to contract the muscles of the trunk and lower extremities, especially knee and foot muscles, in order to control body balance and stability. Exercise might positively induce an increase in intra-epidermal nerve fiber branching, resulting in a lessening of pain and diabetic neuropathic symptoms, such as numb feet, burning pain, and a deficit of pressure and vibration perception.[37] Balducci et al. [15] studied the effectiveness of walking on a treadmill in people with DPN; the results showed that this program could increase nerve conduction velocity of sural sensory nerve and peroneal motor nerve. Moreover, Kluding et al. [37] showed that a 10-week program of aerobic and strengthening exercises could increase cutaneous nerve fibers, leading to the reduction of plantar numbness among people with DPN after the intervention.

The present mini-trampoline exercise program was simple and convenient for use at home. Moreover, monitoring via telephone enabled participants to adhere to the exercise program (38). This type of exercise would be suited to DPN patients who were able to control body balance during standing on an unstable surface. Patients who had poor balance or reduced leg muscle strength should avoid using a mini-trampoline without handrails to reduce the risk of falls. Although the duration of exercise intervention was quite short, it could lead to significant improvements in measures of peripheral neuropathic deficit. The change in neuropathic symptoms might be influenced by changes in vascular function, muscle strength, plantar pressure distribution, or psychosocial factors. From this study, subjects in the exercise group reported that they had smaller calluses, better balance and no further occurrences of foot ulcer. As all subjects in the exercise group were able to complete the intervention without any injuries or complaints, this intervention could be recommended for use in clinical practice to prevent diabetic foot complications. After completion of the exercise program at week 8, all participants were not allowed to continue the exercise program on mini-trampoline until week 20. However, if they were interested to continue the exercise program after week 20 follow-up, they were allowed to consult with the researcher to continue and progress the level of the exercise program. The specific recommendation was patients should avoid exercise on a mini-trampoline if they experienced hypoglycemic symptoms such as dizziness, weakness, or difficulty concentrating. Also, they should regularly examine their feet before and after performing the exercise.

The limitations of the current study were related to the use of a small sample size and convenience sampling method. Those methodological limitations must be kept in mind when interpreting the results. To show significant improvements in outcome measures, a larger sample size would be needed. There were some obstacles in carrying out the current study. The study recruited patients with DPN who were able to balance on a mini-trampoline; however, it was difficult to obtain patients who met this criterion by the commencement of the study. Although the main researcher proposed a random sampling method when designing the research protocol, some potential subjects declined to exercise on a mini-trampoline due to fear of falling. This made it impossible to randomize subjects into groups. Subjects allocated into the trampoline group were those who agreed to participate and who were ready to perform the exercise program regularly. At the end of the 8-week intervention, all subjects still followed the exercise program, indicating good patient compliance to the mini-trampoline exercise. Meanwhile, two subjects dropped out of the control group for unknown reasons. The principal limitation of the convenience sampling method is that it allows the possibility for bias to skew the results of the study. However, baseline general characteristic data were tested and revealed no statistically significant difference between groups at the initial phase.

The mini-trampoline exercise program might not be suitable for applying to other groups of patients with DPN, especially patients who were unable to control themselves on an elastic surface. Although no accidents or injuries were reported by subjects in the exercise group, future research should improve the safety of the mini-trampoline application. Moreover, this exercise program might not be appropriate for those patients with past history of ulceration, especially those who have already had foot deformity, because they might have difficulty in maintaining both static and dynamic balance.

## Conclusion

The aim of this study was to investigate the effects of a weight-bearing exercise program, using a mini-trampoline, on 1st MTPJ ROM, peak plantar pressure and sensation perception in patients with DPN. The results showed that the program could decrease the feeling of numbness, and the number of people with DPN with impaired pressure and vibration perception within a relatively short period of time. Additionally, the exercise could increase 1st MTPJ flexion and extension. There was a trend for a reduction of peak plantar pressure in the medial forefoot of exercising subjects. It is feasible that a simple home exercise program using a mini-trampoline could provide the benefits of fewer foot ulcerations in people with DPN.
